# Importance of the Wrist Extensor Muscle Training: Two Cases of Elbow Flexorplasty following Traumatic Brachial Plexus Injuries

**DOI:** 10.1155/2018/4691796

**Published:** 2018-05-16

**Authors:** Shigeki Kubota, Tadashi Kubo, Hiromi Kameda, Yoshiyasu Itoh

**Affiliations:** ^1^Department of Rehabilitation Medicine, Keiyu Orthopaedic Hospital, 1741 Hanetsuku, Tatebayashi, Gunma 374-0011, Japan; ^2^Department of Orthopaedic Surgery, Keiyu Orthopaedic Hospital, 1741 Hanetsuku, Tatebayashi, Gunma 374-0011, Japan

## Abstract

The modified Steindler procedure—a reconstructive surgery used to restore elbow flexion following upper brachial plexus injuries—involves shifting the origins of the muscle groups responsible for wrist flexion and forearm pronation originating from the medial epicondyle to the proximal direction to be used as flexors of the elbow. In the postoperative rehabilitation, we focused on strengthening not only the transferred muscle but also the wrist extensor muscles as antagonist muscles. After reconstruction surgery for elbow flexion via the modified Steindler procedure for traumatic brachial plexus injury, we performed long-term rehabilitation to strengthen the antagonist muscles. As a result, in two cases, excellent elbow flexion strength and gripping strength were achieved, confirming the importance of the antagonist muscles.

## 1. Introduction

Brachial plexus injury is one of the most serious peripheral nerve injuries resulting in upper limb dysfunction commonly due to trauma in motorcycle accidents. The Steindler procedure is a classical reconstruction surgery to restore flexion of the elbow in upper type of brachial plexus injuries in which the origins of the wrist flexor muscle groups are transferred to the proximal direction [1]. Leo Mayer modified the original Steindler procedure to prevent pronation contracture by shifting the muscle-tendon transplant more anteriorly [2]. However, the proximal-radial transposition was restricted because the ulnar head of the flexor carpi ulnaris (FCU) was left in situ. We mobilized the ulnar head of the FCU en bloc with the flexor-pronator muscle group originating from the medial epicondyle and the median and ulnar nerves with their concomitant vessels. Therefore, increasing the strength of the muscles of wrist flexion also increases the strength of elbow flexion. However, if the strength of the wrist extensor muscles (antagonist muscles) is insufficient during elbow flexion by the transferred muscle, the elbow does not flex, but the wrist is flexed [3]. There are only a few reports on the importance of strengthening of the wrist extensor muscle before and after the modified Leo Mayer procedure [4, 5]. However, to date, there have been no reports discussing the importance of rehabilitation of the wrist extensor muscles by focusing on the wrist extensor muscles as antagonists for elbow flexorplasty after brachial plexus injury. In this type of elbow flexorplasty, conservation of the wrist extensor muscles is essential [6], and strengthening these muscles is an important element in increasing the strength of elbow flexion.

In the present study, we implemented a rehabilitation protocol focusing on strengthening the wrist extensor muscles along with the strength of the transferred muscles. We report the results of two patients who achieved excellent outcomes of elbow flexion strength following the reconstruction surgery.

## 2. Case Presentation

**Figure 1:**
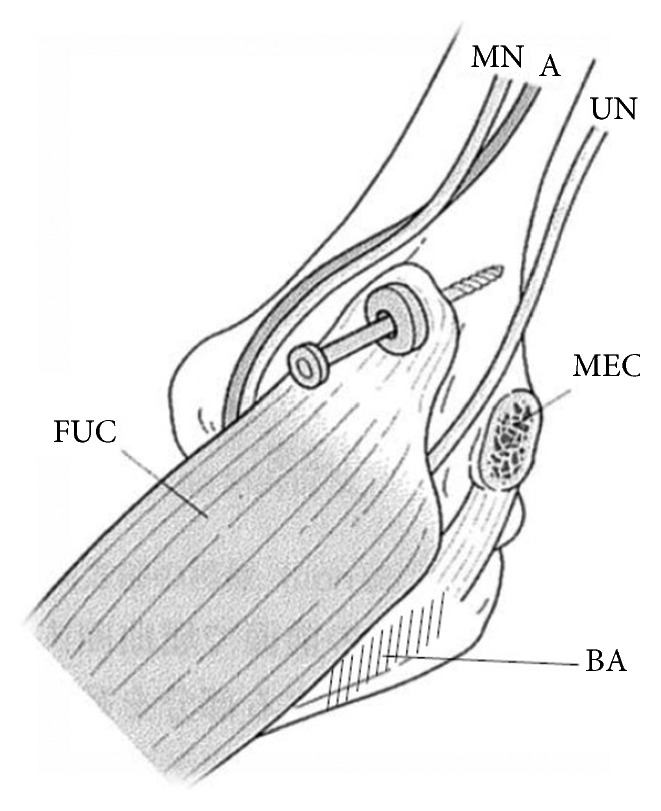
Modification of the Steindler–Leo Mayer procedure (modified SLM procedure) [7]. The flexor-pronator muscle group of the forearm was lifted with the neurovascular bundle along with a bone fragment from the medial epicondyle of the humerus and shifted 8–10 cm to the central and radial sides to act as the primary source of power for elbow flexion. We modified the Leo Mayer procedure to improve the muscle strength further, and not only the humeral head of the FCU but also the ulnar head was detached from the olecranon with the neurovascular bundle. This figure was reprinted from reference [7] by permission of Nankodo Co., Ltd. UN: ulnar nerve; MN: median nerve; A: brachial artery; MEC: cut end of the medial epicondyle; FCU: entire flexor carpi ulnaris muscle and forearm flexor muscle groups; BA: bare area of the ulna after ulnar head of the FCU was mobilized.

Two patients underwent reconstruction for elbow flexion following traumatic brachial plexus injuries between 2008 and 2011. End results were evaluated at 10 months postoperatively. They underwent the above-mentioned improved modification of the Steindler–Leo Mayer procedure [7] (modified SLM procedure: Figure 1). The ages of patients at the time of the surgery were 20 and 57 years, and their postoperative follow-up periods were 10 and 12 months, respectively. Their elbow flexion strength, active range of elbow flexion, and strength of the wrist extensor muscles were regularly examined postoperatively.

### 2.1. Case 1

**Figure 2:**
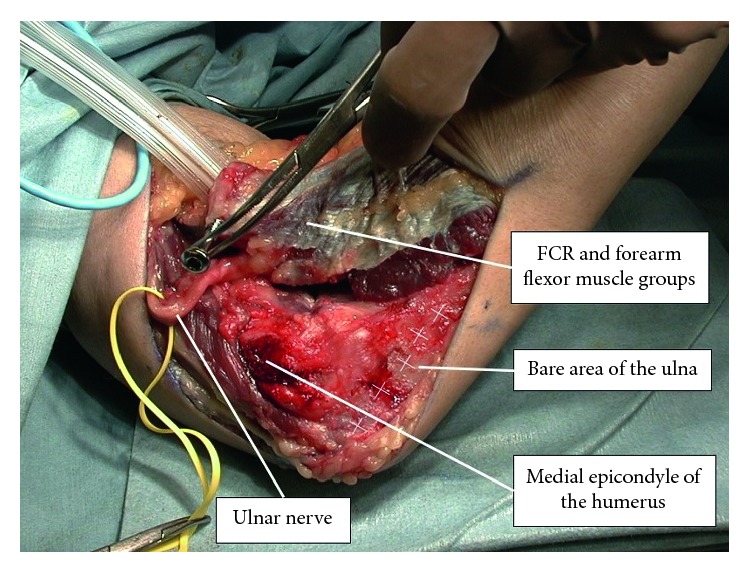
Surgical findings of the modified SLM procedure (Case 1). Whole FCU and forearm flexor muscle groups are lifted with the bone fragment from the medial epicondyle of the humerus and are shifted to the central and radial sides of the humerus. Note the ulnar and median nerves (hidden) protected with a silicone tape.

**Figure 3:**
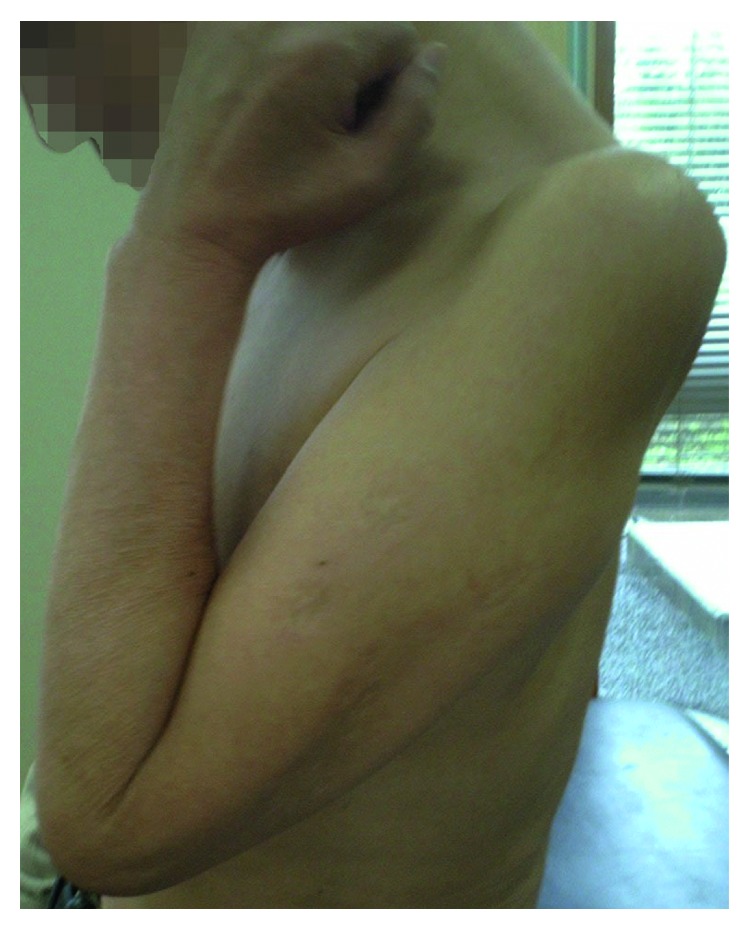
Excellent elbow flexion strength and range of motion at 10 months postoperatively (maximum range of elbow flexion, 140°) in Case 1.

**Figure 4:**
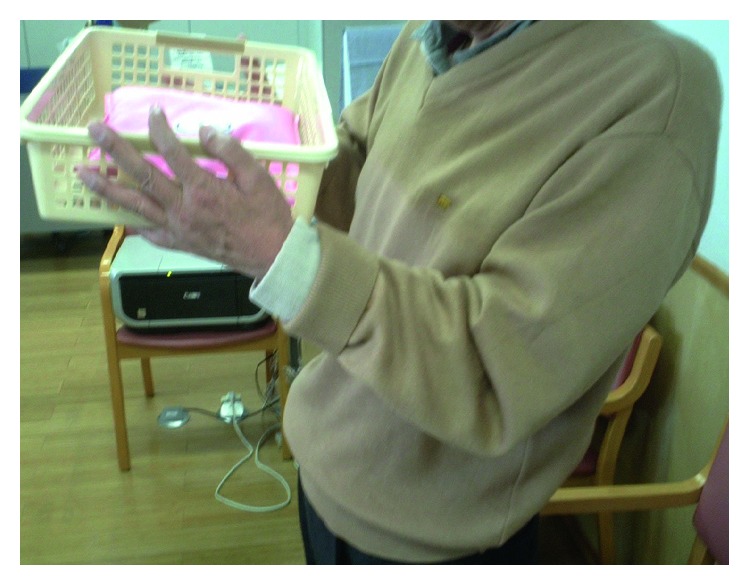
The patient was able to carry a 5 kg object with both hands at 10 months postoperatively (Case 1).

A 57-year-old right-handed male office worker incurred left brachial plexus injury following an automobile accident. He sought medical attention at our hospital four weeks after the injury. Manual muscle testing (MMT) at the initial examination revealed the following muscle strengths on the left side: deltoid, 0; biceps brachii, 0; triceps brachii, 2; wrist flexor muscles, 3; wrist extensor muscles, 3; lumbrical muscles of the hand, 4; and interosseous muscle, 4. Grip strength on the affected side was 6 kg, which was 38% of that on the unaffected side. Electrophysiological examination revealed a denervation potential in the deltoid, biceps brachii, and infraspinatus muscles, and a muscle action potential was confirmed in the serratus anterior muscle. Sensory examination revealed numbness and mild amblyopia at C5 and C6 levels. Assessment of activities of daily living (ADL) was performed with the use of the unaffected limb. During preoperative rehabilitation, we performed the range of motion (ROM) exercises of the joint, strengthening of the remaining muscles, and low-frequency stimulation therapy for the denervated muscles, while hoping for natural recovery of the damaged nerves. The preoperative MMT of the biceps brachii, a reflection of elbow flexion strength, was one. Eight weeks after the injury, we initiated rehabilitation for the patients to strengthen the muscle groups involved in wrist flexion and extension, in order to aid reconstruction of elbow flexion. We chose to perform the modified SLM procedure because his biceps brachii recovered to MMT 1, and the nerve transfer procedure (intercostal nerve transfer) has shown poor results in cases over 40 years [8]. In contrast, we were confident that the modified SLM procedure would provide good and stable results. Seven months following the injury, the modified SLM procedure was performed (Figure 2). After immobilization with a cast for eight weeks, the patient began active ROM exercises for elbow flexion in a gravity-eliminated position and performed active training of the muscle groups responsible for wrist extension. Five months postoperatively, we began the manual resistance exercise, and a month later, we began muscle training using a weight (250 g). Elbow flexion strength at six and ten months postoperatively was 2 and 4, respectively. With the arms hanging at the side, the patient's active range of elbow flexion was 0° preoperatively, which increased to 80° six months after the surgery and 140° ten months after the surgery (Figure 3). Active ranges of elbow extension, forearm pronation/supination, and wrist flexion/extension at ten months after surgery were −20°, full range, and full range, respectively. The preoperative MMT of the muscle group of wrist extension was 3, and it increased to 4 and 5 at six and ten months after the surgery, respectively. At ten months postoperatively, movements such as holding documents with both hands became possible, and the patient was able to return to his work. The patient was also able to carry a 5 kg object with both hands (Figure 4).

### 2.2. Case 2

**Figure 5:**
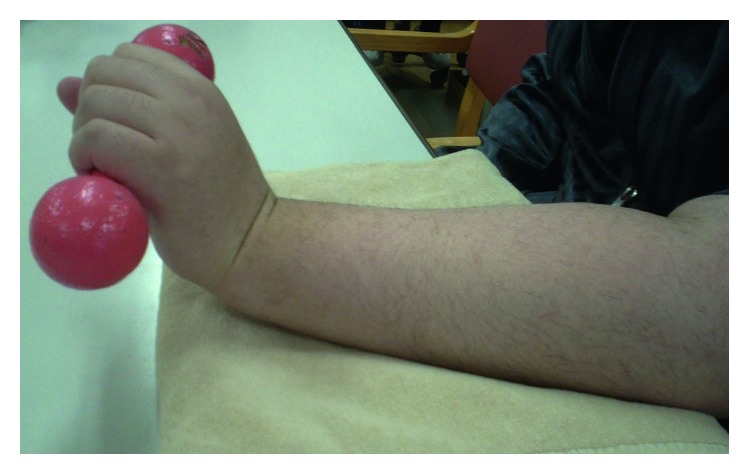
The patient was able to extend the wrist during muscle training using a weight of 1 kg at 12 months postoperatively (Case 2).

**Figure 6:**
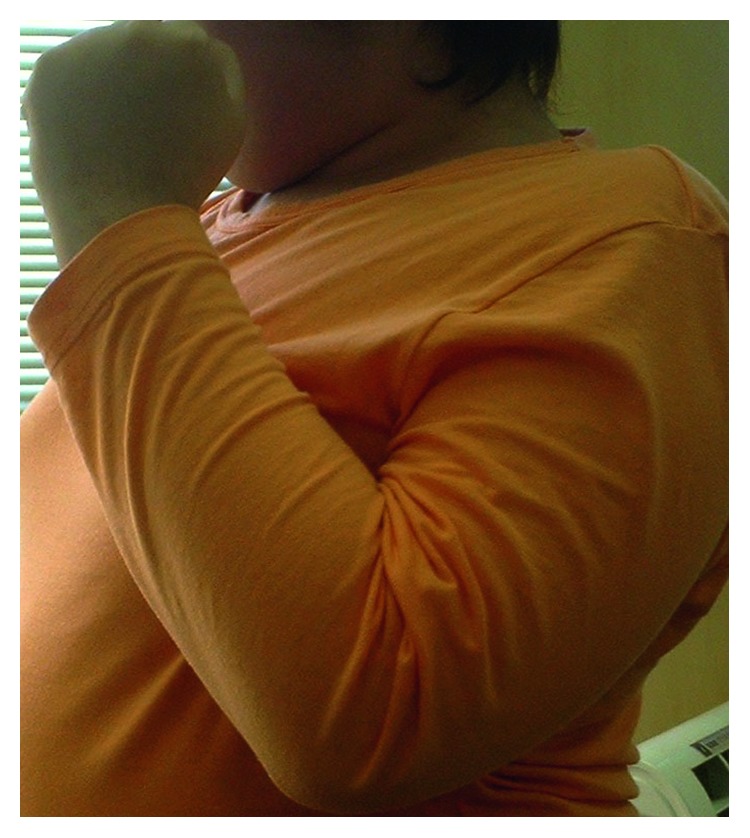
Excellent elbow flexion strength and range of motion of elbow flexion at 6 months postoperatively (maximum range of elbow flexion, 120°) in Case 2.

A 20-year-old left-handed male college student incurred left brachial plexus injury in a motorcycle accident. He sought medical attention at our hospital two weeks after the injury. However, the rehabilitation did not begin until four months after the injury. MMT at the beginning of the rehabilitation on the affected side was as follows: deltoid, 2; biceps brachii, 2; triceps brachii, 4; wrist flexion muscles, 4; wrist extension muscles, 4; lumbricals, 4; and interosseous muscle, 4. His sensory function was only impaired in the region innervated by the axillary nerve. The assessment of ADL performance revealed that he was fully independent with the use of the unaffected limb. The preoperative rehabilitation involved ROM exercises and strengthening of the remaining muscles. Since surgical reconstruction of elbow flexion was planned, the focus was on strengthening of the muscle groups involved in wrist flexion and extension (Figure 5). Recovery of the biceps brachii muscle (elbow flexion strength) stopped at MMT grade 2; thus, the modified SLM procedure was performed one year and five months after the injury. After immobilization with a cast for five weeks, the patient began training of the range of elbow flexion with active motions of the transferred muscle with the support of a sling. Simultaneously, the patient underwent active training of the wrist extensor muscles. Subsequently, he underwent active elbow flexion training, and we initiated manual resistance active elbow flexion training at four months postoperatively. At six months postoperatively, the patient achieved excellent elbow flexion strength (MMT 4), and the active range of elbow flexion increased from 0° preoperatively to 120° postoperatively (Figure 6). The patient was able to lift a plastic bottle up to his mouth. At 12 months postoperatively, his elbow flexion strength remained at MMT 4 and the active range of elbow flexion increased to 145°. The preoperative MMT of the wrist extensor muscle group was 4, and it increased to 5 at 12 months postoperatively. Active ranges of elbow extension, forearm pronation/supination, and wrist flexion/extension at 12 months after surgery were −20°, full range, and full range, respectively. As with the patient in Case 1, the patient in this case was also able to carry a 5 kg object with both hands.

## 3. Discussion

The muscle group responsible for forearm flexion and pronation (pronator teres, PT; flexor digitorum superficialis muscle, FDS; flexor carpi radialis muscle, FCR; FCU; and palmaris longus muscle, PL) originates from the medial epicondyle of the humerus and supports the motion of elbow flexion [9]. The modified Steindler procedure, which shifts the origin of these muscles centrally, provides more efficient moment arm and enables the use of the muscle group as the primary motor of the elbow flexion [1, 2]. Although it is greatly influenced by the muscle tone of the origin of the flexor muscles after shifting to the central and radial sides, we believe that the distance of muscle excursion of PT, FCR, PL, FCU, and FDS would be insufficient for complete elbow flexion. To achieve maximum elbow flexion using solely the muscle group that controls wrist flexion, the wrist needs to be extended to increase the muscle tone of these muscles. In elbow flexion with the transferred muscle, the strength of the muscle group of wrist flexion is used for wrist flexion alone and it cannot flex the elbow. Therefore, strengthening of the wrist extensor muscles becomes crucial to fix the wrist in a neutral position towards wrist extension and resist the increased strength of the muscle group of wrist flexion.

In Case 1, the active range of elbow flexion increased from 80° to 140° at six to ten months postoperatively. The strength of elbow flexion increased from MMT 2 to 4, and the strength of the antagonist muscles of wrist extensor muscle also increased from MMT 4 to 5. Thus, it is assumed that elbow flexion was functionally effective (Figure 3). Wrist extension strength in Case 2 increased from MMT 4 to 5 at six to ten months postoperatively along with an increase in the active range of elbow flexion from 120° to 145°. Since the elbow flexion strength remained at MMT 4 during this period, it is assumed that the increased muscle strength of the wrist extensor muscles contributed considerably to elbow flexion strength.

Gilbert et al. reported the Steindler effect as an important complication, which occurs when there is weakness of the wrist extensors, in a series with the longest follow-up in patients with obstetric paralysis who underwent the Steindler procedure [10]. We understand that the Steindler procedure is contraindicated if wrist extensors show weakness. In patients with obstetrical brachial plexus palsy reported in the study of Gilbert et al. [10], pronation contracture might occur because pronation position was maintained for long time. The modified SLM procedure is an operation that causes more proximal-radial transposition than the usual modified Leo Mayer procedure, which is performed to prevent pronation contracture. Therefore, we expected that severe pronation contracture would not occur in our two cases. We implemented training for focusing on strengthening the wrist extensor muscles in pre-postoperative rehabilitation, and we obtained good results. This training aided in preventing the Steindler effect and obtaining the power for elbow flexion.

In the two cases presented above, not only were the wrist flexor muscles strengthened, which enabled elbow flexion, but also the balance between the flexors and extensors was maintained by the increased muscle strength in the antagonist muscles of wrist extension. As a result, the patients achieved complete flexion of the elbow. In both cases, the wrists extended from the neutral position, indicating the possibility that increased strength of the wrist extensor muscles has notable effects on the active range of motion of the elbow and increases the strength of elbow flexion during maximum elbow flexion.

## 4. Study Limitation

The precise contribution of the wrist extensor muscles to elbow flexion is unclear in the two cases in this study. Thus, in future studies, chronological changes in the active range of elbow flexion need to be examined while comparing the outcomes when only the muscle group of wrist flexion was trained versus when it was combined with training of the wrist extensor muscles. The small number of patients is also an important limitation of the study.

## 5. Conclusion

In the two reported cases of traumatic brachial plexus injuries in which reconstructions for elbow flexion were performed using the modified SLM procedure, we implemented a long-term rehabilitation protocol in which the flexor-pronator muscle groups that were moved proximally were strengthened, while focusing on the antagonist muscle group of wrist extension as well. The patients achieved excellent elbow flexion strength and active range of motion without postoperative elbow and pronation contractures, and were able to grip objects with both hands.
